# Cognitive Coping Style and the Effectiveness of Distraction or Sensation-Focused Instructions in Chronic Pain Patients

**DOI:** 10.1371/journal.pone.0142285

**Published:** 2016-04-12

**Authors:** Lisa Fox, Jane C. Walsh, Todd G. Morrison, David O’ Gorman, Nancy Ruane, Caroline Mitchell, John J. Carey, Robert Coughlan, Brian E. McGuire

**Affiliations:** 1 School of Psychology, National University of Ireland, Galway, Ireland; 2 Department of Psychology, University of Saskatchewan, Saskatoon, Canada; 3 Centre for Pain Research, National University of Ireland, Galway, Ireland; 4 Division of Pain Medicine, Galway University Hospitals, Galway, Ireland; 5 Rheumatology Department, Galway University Hospitals, Galway, Ireland; 6 School of Medicine, National University of Ireland, Galway, Ireland; Université catholique de Louvain, BELGIUM

## Abstract

**Aim:**

This study set out to investigate whether cognitive coping strategies that match participants’ preferred coping style effectively reduce pain intensity and situational anxiety in a population of people with chronic pain.

**Method:**

Chronic pain patients (*N* = 43) completed questionnaires on coping style, pain intensity, self-efficacy, and situational/trait anxiety. Participants were classified as Monitors (*n* = 16) or Blunters (*n* = 19) based on their Miller Behavioural Style Scale score. Participants were then provided with an audiotaped intervention in which they were instructed to focus on pain sensations or to engage in a distraction task and then to rate the pain intensity and their anxiety during and after the attentional focus and distraction conditions. The two interventions were each completed by all participants, having been presented in counterbalanced order.

**Results:**

Findings revealed that Monitors’ level of anxiety decreased following a congruent (i.e., sensation-focused) intervention. No effects were obtained in terms of perceived pain. For blunters, however, their perceived levels of anxiety and pain did not attenuate following a congruent, distraction-focused intervention.

**Conclusion:**

Among persons experiencing chronic pain, tailoring coping strategies to match an individual’s preferred coping style–in particular, those with a high level of monitoring–may enhance the benefit of psychological approaches to management of anxiety.

## Introduction

As humans possess a limited capacity for attending to internal and external stimuli, distraction may serve as a useful means of altering perceptions of noxious stimuli such as pain. Further, as the attentional resources consumed by a distracting task increase, one’s ability to process pain diminishes [1].

Unfortunately, the presence of chronic, unremitting pain makes constant distraction virtually impossible, especially if the pain is so severe that it demands attention [2,3]. This observation has led some researchers to propose that focusing on pain sensations may be more beneficial, either through a detached awareness of the symptoms (elucidated in newer mindfulness and acceptance-based interventions) or through a process of examining the pain sensations and reframing them in a way that augments comfort.

The relative merit of distraction and attention-focused interventions has been examined in a meta-analysis [4], whereby the authors suggested that distraction was more effective for acute pain stimuli but attention was more beneficial in the case of a long-term stressor. Two points, however, warrant mention: 1) individual variation exists in the phenomenology of pain; and 2) the bulk of studies on this topic may have limited relevance for chronic pain populations as they utilize pain that is experimentally induced and, thus, of questionable ecological validity. While the relative merits of being distracted from, or attending to, pain sensations remains unresolved, an important variable in the debate is preferred coping style.

The idea that people are predisposed to favour a particular coping style is not new [5]–several authors have used interchangeable terms to categorise coping styles as being essentially an avoiding/distraction style or a confronting/monitoring style [6,7]. Individuals who are described as *Blunters* typically cope with threat by distracting themselves and avoiding threatening cues [8] while *Monitors* are more likely to attend closely to threatening material and seek out additional cues.

Findings from a recent study underscore the utility of matching preferred coping style with the appropriate cognitive strategy in tolerance for laboratory-induced pain in healthy participants [9]. Using the Miller Behavioural Style Scale (MBBS) [8] to examine monitoring and blunting coping styles, the authors [9] found that participants in the matched condition (e.g., monitoring style coupled with a sensation-focused cognitive strategy) reported significantly higher pain thresholds than participants in the mismatched condition (e.g., monitoring style and distraction strategy). The benefits of congruency also were noted by Shiloh and associates [10] who found that, in comparison to their blunting counterparts, women with a monitoring coping style reported less pain whilst viewing a contraction monitor.

The applicability of findings based on matching between coping style and a sensation versus distraction cognitive orientation for people with chronic pain is unclear. Thus, the central aim of the current study is to address this omission. Based on the literature cited, it is hypothesized that matching preferred coping style with the appropriate cognitive intervention strategy would result in reduced pain and situational anxiety in a chronic pain population. A subsidiary purpose is to examine other variables that have been shown to influence the perception of pain, including pain self-efficacy [11–13] and anxiety [14–16].

## Method

### Participants

Forty-three participants with chronic pain were recruited from a tertiary pain management clinic (*N* = 30) and rheumatology clinic (*N* = 13). The inclusion criteria for the study were: (1) aged over 18 years (2) history of chronic pain (3) no major psychiatric or cognitive impairment that would prohibit the capacity to understand the study, complete the questionnaires and give informed consent. In each of the clinics, posters about the study were displayed and the contact details of the first author were provided. When potential participants contacted the first author, the study was explained and if the participant wished to partake and met the inclusion criteria, recruitment proceeded from there. There were 37 women and 6 men, with a mean age of 46.36 (*SD* = 13.1; range = 20 to 74 years). The average amount of time that participants reported experiencing chronic pain was 4.6 years (*SD* = 1.23 years; range = 6 months to 9 years). The most frequently reported sites of pain were back (*n* = 22), neck and shoulder (*n* = 15), and leg (*n* = 10). Thirty one (72.1%) participants had pain in more than one location.

### Procedure

The study received ethical approval from Galway University Hospital and the National University of Ireland, Galway. An informed consent sheet, detailing the study’s purpose as well as participants’ freedom to terminate their involvement at any time without penalty and their rights to anonymity and confidentiality, was distributed and signed.

Participants completed a questionnaire pack and then listened to two audio-recorded interventions. The latter were counterbalanced to control for possible order effects.

### Measures

Demographic information was obtained regarding age, gender, type of chronic pain, parts of the body affected by chronic pain, length of time experiencing chronic pain, and diagnosis (if one had been received).

The Pain Self-Efficacy Questionnaire (PSEQ) [17] is a 10-item measure of an individual’s confidence that, despite chronic pain, he/she can perform a variety of activities. It uses a 7-point Likert scale (0 = not at confident; 6 = completely confident), with total scores ranging from 0 to 60. Higher scores reflect stronger self-efficacy beliefs. The psychometric integrity of the PSEQ has been well-documented [18–21]. For the current study, scale score reliability was .85 (95% CI = .78-.91).

The State-Trait Anxiety Inventory (STAI) [22] is comprised of two 20-item self-report measures. The STAI State questionnaire instructs respondents to indicate how they feel “right now, at this moment” using a 4-point Likert scale (1 = *not at all*; 4 = *very much so)*. The STAI Trait questionnaire measures how respondents “generally feel” and also uses a 4-point Likert scale (1 = *almost always*; 4 = *almost never*). Total scores on both the state and trait measures can range from 20 to 80, with higher scores reflecting stronger levels of anxiety. The psychometric soundness of the STAI has been demonstrated consistently [23]. In the current study, Cronbach’s alpha coefficients were: STAI State (α = .68, 95% CI = .53-.81; STAI Trait α = .92, 95% CI = .89-.95).

The Miller Behavioural Style Scale—Short-Form (MBSS) [8] was used to identify an individual’s attentional processing style. Specifically, individuals may be categorised as higher or lower in monitoring based on how they select, encode, and manage threatening material. Those higher in monitoring tend to scan for, and selectively attend to, threatening material, and often seek out further information to increase their knowledge of the situation. Conversely, persons lower in monitoring often cope with threat by *blunting* or distracting themselves and avoiding threatening cues [8].

When completing the short-form version of the MBSS, participants are given two potentially stress-provoking scenarios: visiting the dentist’s office and the threat of job loss. For each scenario, participants select from among eight different options the ones that best apply to them in that hypothetical situation. Four of the options denote monitoring (e.g., “I would ask the dentist exactly what work was being done”) and four reflect blunting (e.g., “I would take a tranquilizer or have a drink before going”). A dichotomous response format (i.e., yes/no) is employed. For the dentist scenario, Cronbach’s alpha coefficients for the monitoring and blunting subscales were suboptimal: .55 and .34, respectively. Scale score reliability for the job loss scenario, however, was acceptable: .69 (monitoring) and .62 (blunting). Thus, for all subsequent analyses, only the job loss scenario was utilized.

The number of monitoring options that are selected for the job loss scenario are subtracted from the number of blunting options that are chosen. Thus, total scores could range from -4 to +4 (higher positive values suggest a stronger propensity for monitoring). Previous research has confirmed the validity of the MBBS in medical situations [24] and has shown that coping preferences are stable over at least a 4-month period [8].

Pain and Anxiety Numerical Rating Scales (NRS) were also utilized. Participants were asked to estimate their degree of current pain using a NRS that ranged from 0 (“no pain”) to 10 (“worst pain imaginable”). A similar measure was employed to assess anxiety (0 = “completely at ease”; 10 = “extremely anxious”). Such measures are widely used and have been shown to be reliable, valid and appropriate for use in clinical practice [25].

### Interventions

The two interventions consisted of audio scripts that participants listened to via headphones. The scripts were based on excerpts from two widely used pain treatment manuals: “Psychological approaches to pain management: A practitioners’ handbook” [26] and “Pain and behavioural medicine: A cognitive-behavioural perspective” [27]. A clinical psychologist with expertise in the topic of pain management confirmed the clinical integrity of the scripts as either distraction- or sensation-focused. The interventions lasted approximately four minutes each.

The sensation-focused script asked the participant to focus on his or her pain sensations, but to label them in an objective, non-threatening way. In contrast, for the distraction script, the participant visualised an interactive scene in which she/he was required to focus closely on the description of different sights, sounds, and smells unrelated to pain (see Appendix A).

Participants were asked to retrospectively rate their pain and anxiety on the NRS during each intervention and to rate their pain and anxiety immediately following the intervention.

## Results

The dataset for the study can be accessed by contacting the corresponding author.

For the MBSS, it is customary to categorize individuals as Blunters or Monitors based on a median split of the summary difference score [28]. However, median splits are seldom appropriate [29], thus, an alternate means of classifying participants was used whereby those with negative summary difference scores were classified as Blunters (*n* = 19); those with positive summary difference scores were classified as Monitors (*n* = 16); and those with difference scores of zero were removed (*n* = 8).

Means, standard deviations, and score minima/maxima for all variables are provided in Table 1. Participants’ levels of current pain and anxiety as well as pain self-efficacy were modest (i.e., mean scores on these indicators did not differ significantly from scale midpoints). Levels of trait and state anxiety, as measured by the STAI, also were modest. Further, for Blunters only, the difference between their reported level of trait anxiety and the STAI midpoint was statistically significant, *t* (18) = -3.08, *p* = .006.

**Table 1:** Means, Standard Deviations, and Score Minima/Maxima for Key Variables.

	*M*	*SD*	Minimum	Maximum
PSEQ	34.49	11.94	5	53
Blunters	35.42	11.54	6	51
Monitors	29.88	11.97	5	53
STAI–State	47.54	7.02	23	72
Blunters	49.38	6.95	40	72
Monitors	46.25	7.29	23	53
STAI–Trait	44.37	11.55	21	70
Blunters	41.89	11.47	25	70
Monitors	45.68	11.38	21	63
NRS–Current Pain	5.49	2.42	0	10
Blunters	5.68	2.50	0	10
Monitors	4.94	2.14	1	10
NRS–Current Anxiety	4.58	2.55	0	10
Blunters	4.58	2.76	0	10
Monitors	4.19	2.46	0	7

Note. PSEQ = Pain Self-efficacy Questionnaire; STAI = State Trait Anxiety Inventory; NRS = Numerical Rating Scale. For all variables, higher scores denote more of the construct. The first row pertains to all participants (*N* = 43). The second and third rows pertain to blunters (*n* = 19) and monitors (*n* = 16). No statistically significant differences were noted between blunters and monitors (*p*s = .17–.66). Effect sizes ranged from |.15–.47|.

Correlations were computed among the variables listed in Table 1 for Blunters and Monitors separately. For Blunters, two statistically significant correlations were observed. Specifically, participants’ 1) current level of pain, as measured by the NRS, was positively associated with their current level of anxiety (also as measured by the NRS), *r* (17) = .46, *p* = .045; and 2) current level of anxiety was positively associated with scores on the measure of trait anxiety, *r* (17) = .61, *p* = .006. Two statistically significant correlations also were observed for Monitors: 1) their current levels of pain and anxiety, both as measured by the NRS, were positively inter-correlated, *r* (14) = .57, *p* = .021; and 2) their level of trait anxiety was inversely associated with their reported pain self-efficacy, *r* (14) = -.65, *p* = .006.

A series of independent samples t-tests then were conducted to determine whether participants classified as Monitors versus Blunters differed in terms of current (i.e., baseline) pain, current (i.e., baseline) anxiety, state/trait anxiety, pain self-efficacy, and duration of chronic pain. No statistically significant group differences were identified (i.e., *t* values ranged from -.98 to 1.39; *p*s range from .17–.76). Given these null findings, baseline NRS pain and anxiety, pain self-efficacy, trait anxiety and state anxiety did not need to be treated as covariates. Consequently, no further consideration of these variables was given.

To test the study’s central hypotheses, the independent variables of interest were type of intervention (distraction-focused/sensation-focused) and coping style (Monitor/Blunter). The dependent variables were self-reported levels of pain and anxiety.

2 x 5 ANOVAs were conducted for both self-reported pain intensity and anxiety, with coping style (Monitor/Blunter) as the between-subjects variable and measurement period (baseline, during the sensation-focused intervention, following the sensation-focused intervention, during the distraction-focused intervention, and following the distraction-focused intervention) serving as the within-subjects variable. These analyses permitted us to determine whether a match between preferred coping style and type of cognitive intervention (i.e., distraction- versus sensation-focused) would result in lower levels of pain and situational anxiety, as measured by the NRS, than would a mismatch between coping style and intervention.

For the first ANOVA, a matching effect would be evident if those classified as Monitors reported the lowest levels of pain during the sensation-focused intervention. In contrast, Blunters should report the lowest pain when distracted. Mauchly’s test of sphericity was statistically significant χ^2^ (9) = 45.30, *p* < .001; thus, the Greenhouse-Geisser correction was employed. There was no main effect for measurement period on self-ratings of pain, *F* (2.54, 83.85) = 1.50, *p* = .23, *η*^*2*^ *=* .04, suggesting that levels of pain did not differ significantly across the five measurement points. There also was no statistically significant main effect of coping style on self-ratings of pain, *F* (1, 33) = 1.63, *p* = .21, *η*^*2*^ *=* .04. Finally, no statistically significant coping style X measurement period interaction emerged, *F* (2.54, 83.85) = 2.23, *p* = .10, *η*^*2*^ *=* .06, which was contrary to the proposed matching effect. (See Table 2 for mean self-report ratings of pain across the five measurement periods, stratified by Monitor/Blunter coping style.)

**Table 2:** Pain and Anxiety NRS mean (SD) scores for Monitors and Blunters before, during and after each intervention.

	Monitors (*n* = 16)	Blunters (*n* = 19)
**Time 1**	4.50 (1.93)	6.05 (2.46)
	4.19 (2.46)	4.57 (2.76)
**Time 2**	4.44 (1.82)	5.68 (2.26)
	2.00 (1.51)	3.89 (2.51)
**Time 3**	4.69 (2.52)	4.89 (2.51)
	2.00 (1.51)	4.26 (2.64)
**Time 4**	4.63 (2.13)	5.42 (2.36)
	3.25 (2.11)	2.84 (2.73)
**Time 5**	4.94 (2.14)	5.68 (2.50)
	3.44 (1.86)	3.11 (2.87)

Time 1 = Baseline, Time 2 = During sensation-focused task, Time 3 = Immediately after sensation-focused task, Time 4 = During distraction task, Time 5 = Immediately after distraction. For each time period, top entries (e.g., 4.50 and 6.05) refer to pain intensity ratings whereas bottom entries (e.g., 4.19 and 4.57) refer to anxiety ratings.

For the second ANOVA, a matching effect would be demonstrated if the lowest levels of anxiety were reported by Monitors during the sensation-focused intervention and by Blunters during the distraction intervention. As Mauchly’s test of sphericity was statistically significant, χ^2^ (9) = 83.09, *p* < .001, the Greenhouse-Geisser correction was used. No statistically significant main effect was obtained for coping style (Blunter versus Monitor) on self-reported levels of anxiety, *F* (1, 33) = 1.56, *p* = .22, *η*^*2*^ *=* .05. A statistically significant main effect for measurement period on self-ratings of anxiety was found, *F* (2.27, 74.86) = 3.92, *p* = .02, *η*^*2*^ *=* .11, which, in turn, was qualified by a significant coping style X measurement period interaction, *F* (2.27, 74.86) = 4.45, *p* = .012, *η*^*2*^ *=* .12 (see Table 2). Planned comparisons revealed that, as predicted, anxiety levels reported by Monitors (*M* = 2.00, *SD* = 1.51) were significantly lower than anxiety levels reported by Blunters (*M* = 4.26, *SD* = 2.64) following the sensation-focused intervention, *t* (29.32) = 3.17, *p* = .004, *d* = 1.05. However, no difference in level of anxiety was evident between Monitors (*M* = 3.44, *SD* = 1.86) and Blunters (*M* = 3.11, *SD* = 2.87) following the distraction intervention, *t* (33) = -.40, *p* = .69, *d* = .14. Also, for Monitors, their level of anxiety was significantly lower following the sensation-focused intervention (*M* = 2.00, *SD* = 1.51) than following the distraction-focused intervention (*M* = 3.44, *SD* = 1.86), *t* (15) = -3.94, *p* = .001, *d* = -.85. In contrast, Blunters’ level of anxiety did not change significantly during these same time periods (i.e., after the sensation-focused [*M* = 4.26, *SD* = 2.64] versus after the distraction-focused [*M* = 3.11, *SD* = 2.87] intervention, *t* [18] = 1.77, *p* = .09, *d* = .42). These findings suggest that the influence of congruency between coping style and type of intervention may be more pronounced for Monitors than Blunters in terms of anxiety reduction.

**Fig 1:**
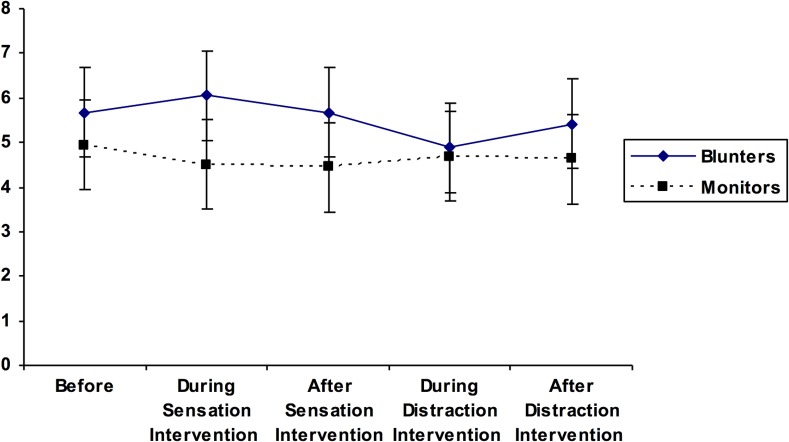
Pain ratings across conditions.

**Fig 2:**
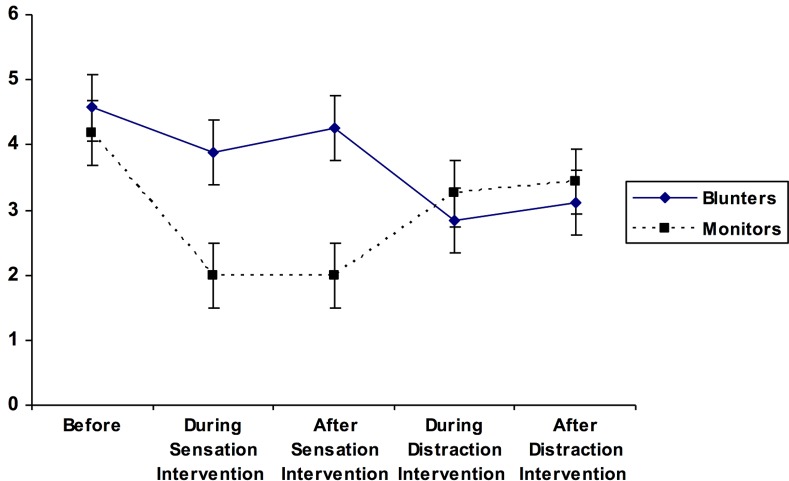
Anxiety ratings across conditions.

The findings are summarized in Figs 1 and 2, below, showing the pain and anxiety ratings for the two groups across each of the conditions.

## Discussion

There is now mounting evidence that pain and cognition are linked: cognitive functioning affects pain perception and pain impacts on cognition [30]. Attention is a key cognitive mechanism for either magnifying or reducing awareness of pain [31]. Previous research suggests that distraction strategies may interrupt the processing of nociceptive stimuli [32] and sensation-focused approaches may alter the affective interpretation of pain [33]. Our results offer some support for the hypothesis that matching one’s preferred cognitive coping style with the appropriate cognitive strategy has a beneficial effect on pain intensity and situational anxiety. Monitors had significantly lower levels of anxiety during and after the sensation-focused task; however, a similar effect did not emerge for Blunters (i.e., their level of anxiety did not decrease following a distraction-focused intervention). As well, neither Monitors nor Blunters evidenced statistically significant changes in perceived pain following either type of intervention.

In contrast, a similar study [9], which induced pain by a cold-pressor in healthy participants, found no effect on anxiety. How might the discrepancy between this research and the findings we report be resolved? One possible explanation resides in the type of pain being investigated: laboratory-induced pain does not have the same long-term negative impact and is not as emotionally threatening as chronic pain [34]. Consequently, the anxiety components of studies examining acute versus chronic pain may not be comparable. The current study’s use of persons experiencing chronic pain also may account for our inability to replicate the pain intensity effects noted by researchers examining induced pain [9]. Arguably, chronic pain is experienced by patients as a constant reminder of their condition and its malleability vis-à-vis short-term interventions may be limited [35]. For example, a recent study [36] compared 10 weekly, individual, 60-minute sessions of CBT and found that, despite improvements in other areas, mean pre- and post-treatment scores on the numeric pain rating scale did not differ significantly despite this intensive intervention.

The study had a number of limitations. Firstly, a heterogeneous pain sample was utilized. It is possible that the findings were influenced by part(s) of the body affected, and presence or absence of a diagnosis or the influence of disease status (e.g. in rheumatic patients). Another consequence of employing a heterogenous sample is that the sensation-focused task was necessarily generic rather than specific to site of pain. Furthermore, male participants were under-represented relative to women, although this is consistent with the general population. However, several studies suggest differences in both pain threshold and tolerance between men and women, with women demonstrating a greater sensitivity than men [37]. Future studies should seek to examine the possible influence of gender on outcomes. We did not check with participants whether they had actually applied the respective strategies although we contend that it is likely they did apply the strategies because they were listening directly to the scripts whilst wearing headphones. However, we suggest that future studies should incorporate a validity check to determine if the interventions were actually utilized by the participants.

The reliability of the MBBS Monitor and Blunter sub-scales was found to be modest and previous research on which the current experimental design was based [9] used a Total Monitoring score to assign participants to preferred coping style as they noted that the monitoring scale has been shown to have higher scale score reliability than the blunting scale. As yet, it is unclear which method of assigning individuals to preferred coping style is most accurate, but this warrants consideration in future studies.

In order to minimize the time demands placed on participants, it was necessary to construct brief, once-off interventions. However, an extended intervention may have yielded different results as almost all psychologically-based coping strategies require practice and repetition to become “ingrained” as coping responses. In addition, it has generally been suggested that sensation-focused strategies such as mindfulness meditation have a cumulative effect when practiced over time [38]. This may also be the case for distraction-based interventions. Future research should evaluate the comparative effectiveness of these interventions when rehearsed frequently over an extended period. Such interventions have the benefit of being brief, portable and cheap and so any benefit derived for patients, even of a modest scale, is considered clinically useful.

Another possible limitation pertains to the unestablished reliability and validity of the interventions employed. Although they were obtained from widely-used treatment manuals and were assessed by a psychologist experienced in the treatment of chronic pain, the interventions were novel. Thus, it may be beneficial for future researchers to have more than one expert confirm the validity of the interventions.

Other variables that were not controlled as part of this study may also be influential. For example, the motivational context of attentional deployment may be important–in other words attentional processing of pain information will be particularly enhanced when the focal goal is related to pain management whereas this may not be the case if the focal goal is unrelated to pain [31,39]. An experimental study examined pain catastrophizing, which is known to have a powerful influence over response to pain, and the presence or absence of a motivation (reward) to engage in distraction. The study showed that increasing the motivational relevance of the distraction task increased the effects of distraction, especially for those who catastrophized about pain [40]. In another study, high catastrophizers achieved little distraction-related analgesia early in a session of experimentally-induced pain but achieved pain reduction later–thus, a tendency to catastrophize may somehow delay the deployment of attention diversion or initially over-ride the beneficial effects of distraction [10]. Future extensions of the current study should, therefore, examine potentially important variables such as motivation and catastrophizing. In fact, it is possible that coping strategy may be most influential when applied to pain-related goal conflicts, such as the avoidance of pain versus pursuit of a reward [10]. The fear-avoidance model of Vlayen and Linton [41] would provide a good vehicle by which to empirically test such questions. Finally, sample size and power calculations for our study were based on similar studies in this area [42]. Effect sizes observed were mainly medium which suggests that the present sample size, whilst adequate, could have been increased slightly.

In conclusion, our results tentatively suggest that customizing treatment in accordance with the patient’s preferred coping style may achieve the most favorable outcomes. This was especially so for current anxiety among persons classified as Monitors and exposed to a congruent intervention (i.e., sensation-focused). Future research should seek to replicate and extend these findings with specific chronic pain conditions.

## Appendix A

Intervention 1 –Sensation-Focused Script

“*I want you to think about your pain for a few moments*. *Imagine that you can see your pain from a great distance away*. *Do not think about how much it hurts*, *but just focus on describing it to yourself*. *Now*, *go to the pain’s location*. *Explore the pain and be open to the sensations that you are feeling*. *Where are the sensations coming from*? *Are they radiating from within your body or are coming from your body’s surface*? *Does the pain come and go*, *rising and falling like a wave*? *Or is it more of a dull pain*, *like being under a weight that is putting pressure on your body*? *Maybe the pain is more of a knotted feeling inside your body or a hot or sharp sensation*? *Now*, *think about changing the way you see the pain*. *If the pain appears as a knot*, *I want you to try to imagine untangling the knots*, *pulling them apart*, *one by one*, *until there are none left*. *If the pain feels hot and sharp*, *think about the edges of the pain becoming more rounded and gentle*, *try to see the edges softening*. *Think of the heat of the pain reducing to a comfortable warmth*, *like warm sunshine*. *Think about cooling down the pain further—imagine blowing cool arctic air on the pain Now imagine that the pain has a shape*. *Imagine that the tightness in your body is like a balloon; see the balloon filled with pressure*. *You can feel the pressure in your body like the air pressing and expanding tight against the balloon*. *The air has a colour*, *what colour is that pressurised air*? *Any colour is fine*, *just notice what colour comes to mind*. *Now*, *you can begin to let the air out of the balloon and you can breathe out the tension*. *Listen to the sound as the air moves out*, *the pressure drops*. *Notice the air as it leaves the balloon in the same way that the sensations in your body can blow out into the air*, *releasing the pressure you feel*. *Now*, *try to see the pain as a smaller*, *weaker*, *gentler picture–try to hold the new picture in your mind for a few moments*. *Whenever you are ready*, *you can bring this image to a close*.”

Intervention 2 –Distraction Script

“*Now*, *I am going to describe some images*, *I want you to try and involve yourself in the images in order to make them as vivid as possible*. *Let’s begin*:

*Imagine a pure white plate with a lemon on it*, *resting on a table*. *See the glossy yellow of the lemon’s skin against the whiteness of the china plate*. *Notice the texture of the lemon as you touch it lightly with your fingertips*. *The fruit’s skin feels smooth and waxy*. *The lemon looks clean and fresh*. *There is a knife on the table*, *next to the plate*. *The knife has a wooden handle and its blade looks sharp and gleaming*. *Now*, *imagine that that you’re picking up the knife*. *You hold the lemon on the plate with one hand*, *and with the other*, *using the knife*, *you cut the lemon in two*, *hearing the knife cut through the lemon and hit the plate*. *As the sharp edge slices into the lemon*, *the juice runs out onto your fingers and onto the plate*. *Notice the stickiness of the liquid as it pours over your fingers*. *The citrus odour immediately hits your nose*: *sharp*, *clean*, *pungent*, *delicious*, *and invigorating*. *Now you pick up one of the lemon halves*, *with the juice still dripping onto your fingers and onto the plate*. *Using the knife again*, *you cut a wedge from the lemon half*, *raise the wedge to your mouth*, *and touch your tongue against it gently*. *Every taste bud in your tongue is drenched with the tangy lemon juice as your mouth puckers instinctively*. *A shiver goes up and down your spine*, *and your shoulders shake*. *Then you pick up the lemon half again*, *and with the knife you cut another wedge*, *the juice continues to drip onto your fingers and out onto the plate*. *You notice that there is also a glass of water on the table*. *The glass contains some fizzy water and there are two ice cubes in the glass*. *Now imagine that you drop the lemon wedge into the glass of water*. *Notice how the bubbles rush to the surface*, *and the crackling noise the bubbles make*. *As the crackling of the bubbles becomes quieter*, *you slowly pick up the glass and put its edge to you lips*. *As you tilt your head back the water rushes into your mouth*. *The water is cool and refreshing*, *notice the bitter citrus flavour as you swallow the water and the tingling sensations of the bubbles on your tongue*. *Then with one more gulp you drain the contents of the glass*. *Now return the glass to its resting place and you begin to move away from the table*. *Picture for a moment the lemon*, *the cutting*, *the tasting*, *the smells……*.*Whenever you are ready you can bring this image to a close*.”
